# Lubrication and Wear Characteristics of Mechanical Face Seals under Random Vibration Loading

**DOI:** 10.3390/ma13061285

**Published:** 2020-03-12

**Authors:** Wentao He, Shaoping Wang, Chao Zhang, Xi Wang, Di Liu

**Affiliations:** 1 School of Energy and Power Engineering, Beihang University, Beijing 100083, China; hwtwin@126.com (W.H.); xwang@buaa.edu.cn (X.W.); 2 School of Automation Science and Electrical Engineering, Beihang University, Beijing 100083, China; shaopingwang@buaa.edu.cn (S.W.); zhangchao303@126.com (C.Z.); 3 Beijing Advanced Innovation Center for Big Data-Based Precision Medicine, Beihang University, Beijing 100191, China

**Keywords:** mechanical face seals, wear, random vibration, lubrication regime, dynamic model

## Abstract

The service life of mechanical face seals is related to the lubrication and wear characteristics. The stable analytical methods are commonly used, but they cannot address effects of random vibration loading, which, according to experimental studies, are important factors for lubrication and wear of mechanical face seals used in air and space vehicles. Hence, a dynamic model for mechanical face seals is proposed, with a focus on the effects of random vibration loading. The mechanical face seal in the axial direction is described as a mass-spring-damping system. Spectrum analysis specified for random vibration is then performed numerically to obtain the response power spectral density (PSD) of the mechanical face seal and calculate the root mean square (RMS) values under random vibration conditions. A lumped parameter model is then developed to examine how dynamic parameters such as stiffness and damping affect the lubrication regimes of mechanical face seals. Based on the dynamic model and Archard wear equation, a numerical wear simulation method is proposed. The results elucidated that the increase of input acceleration PSDs, the decrease of axial damping, and the increase of axial stiffness lead to the probability of the mechanical face seal operating under full film lubrication regime increase and finally the decrease of wear. This research provides a guideline for improving the adaptability of mechanical face seals under random vibration environments.

## 1. Introduction

Mechanical face seals are used in rotating equipment such as pumps, mixers, blowers, and compressors. The seal incorporates both rigid and flexible elements that maintain contact at the sealing interface and slide on each other, allowing a rotating element to pass through a sealed case [1,2,3]. A mechanical face seal used in hydraulic pumps is investigated in this paper, with a focus on random vibration loading [4,5].

Hydraulic pumps are widely used in air and space vehicle fuel systems because of their advantages of high effectiveness and fast response. Failure of a hydraulic pump can produce serious consequences and the working environment of the pump is complex, creating enormous challenges for mechanical face seal design. Particularly in aeronautic and astronautic applications, the random vibration effect requires close attention. To achieve a high level sealing performance for mechanical face seals, an investigation of the influence of random vibration on mechanical seals is imperative to enhance the working reliability of hydraulic pumps.

**Figure 1:**
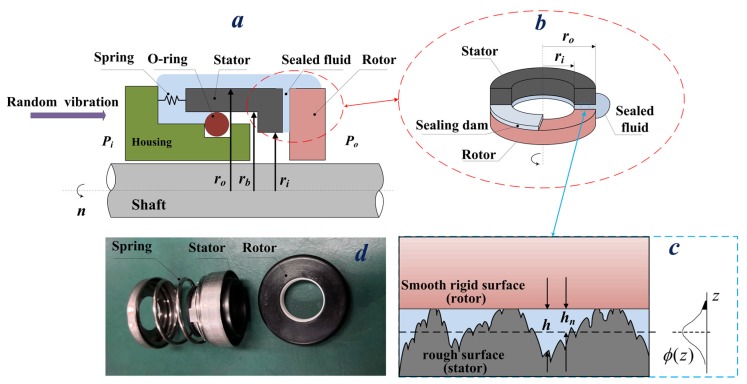
(**a**) Schematic of mechanical face seal; (**b**) sealing dam between the rotor and the stator; (**c**) schematic of asperity contact; (**d**) seal rings.

The main components of a face seal are two rings, the stator, and the rotor, which are arranged perpendicular to the axis of the rotating shaft, as shown in Figure 1. The stator and the rotor keep fit under the force of fluid pressure and compensating mechanism. There are two degrees of freedom in the motion between the stator and rotor, which are the axial motion and the circumferential rotation. The contact between the stator and the rotor is the key to determining the performance of the mechanical face seal [6], and they can run in any of the following three lubrication regimes [7,8].

- Full film lubrication regime: The seal face is separated by a sufficiently thick film and there is no contact between the stator and rotor. In this case, the friction and wear of the seal faces can be significantly reduced, however, fluid leakage could be excessive.
- Boundary lubrication regime: The boundary film is partly discontinuous and there are solid contacts in some areas. Fluid pressure has not developed enough to separate the sealing faces, but it can obviously reduce friction and wear.
- Mixed lubrication regime: This is the most common operation mode in many cases. Under these circumstances, part of the load is carried by mechanical contact, while a majority of the load is carried by fluid pressure. In this regime, friction and wear are relatively moderate and fluid leakage is very low.

Various scholars have explored the dynamics of mechanical seals, including Greenwood and Williamson [9], who presented a contact model based on Hertzian theory. A plastic contact model was then proposed by Pullen and Williamson [10] a few years later. Dayan et al. [11] and Zou et al. [12] simulated the dynamics of mechanical face seals to prevent possible contact between surfaces, and Pustan et al. [13] presented theoretical models and experimental investigations of an internal mechanical seal with oscillating stator. The hydrostatic effect on the mechanical seals with oscillating stator was also examined. Minet et al. [14] analyzed and modeled the morphology of the mechanical seal face, and research by Leishear et al. [15] illustrated that relative vibrations between the stator and rotor affect the fluid film, damage the faces, and decrease the life of the seals. It was also determined that the smaller the axial damping between the two rings, the larger the impact force on the surface.

Random vibration has a significant influence on the axial direction of mechanical seals because it alters the lubricating regimes. Significant efforts have been made to study the random vibration responses of such systems [16,17,18]. Influence analysis under random vibration loading is more complicated than under conventional loading. Many methods have been put forward to facilitate such analysis, including stochastic averaging technique [19], the statistical linearization technique [20], and the equivalent nonlinear system method [21]. Under vibration loading, mechanical face seals are in an unsteady state. Varney [22] analyzed the vibration of a noncontacting mechanical face seal caused by misalignment or imbalance. However, few authors have focused on exploring the influence of external random vibration loading on the mechanical face seals.

The focus of this study is the mechanical face seal used in air and space vehicles under random vibration loading. This study takes the surface roughness into account in the statistical sense from a macro perspective. As a small but unique contribution to this area of research, a spectrum analysis method based on frequency domain, and the root mean square (RMS) analysis method are presented to theoretically investigate the random vibration response characteristics. The research conclusions provide guidance for future robustness design under random vibration loading and to reduce wear in engineering applications.

This paper is organized as follows: Section 1 presented an introduction to the work carried out in this paper. In Section 2, the mathematical model of mechanical face seals under random vibration loading is derived. Section 3 provides the flow of numerical calculation, and numerical experiments and analysis are conducted in Section 4. Finally, Section 5 presents the conclusion.

## 2. Modeling of Mechanical Face Seals under Random Vibration Loading

### 2.1. Description of the Mechanical Face Seal

A typical mechanical face seal configuration is presented in Figure 1a. The stator is flexibly mounted to the housing via two elastic parts: a radial support spring and a viscoelastic secondary seal O-ring. The spring supplies a closing force to the stator and the rotor rotates together with the shaft. The inner and outer radii are denoted as *r_i_* and *r_o_*, respectively, *r*_b_ is the balance radius, and the sealed fluid pressure and atmosphere pressure are *P_o_* and *P_i_*, respectively. Random vibration from the environment is then transmitted to the housing. The nominal surfaces are flat and parallel, as shown in Figure 1b, and a sealing dam is formed between the rotor surface and the stator surface. The stator should be rough, whereas the rotor is smooth, as shown in Figure 1c. Additionally, *ϕ*(z) is the probability density function of the surface height distribution, *h_n_* is the nominal film thickness, and *h* is the local film thickness for rough surfaces. The material used in the rotor is SiC (silicon carbide), and the material used in the stator is resin-impregnated carbon. The seal rings sample are presented in Figure 1d. The fluid used in the mechanical face seal is aviation hydraulic oil, which has a density of 870 kg/m^3^ and a kinematic viscosity of 10 mm^2^/s. Therefore, its dynamic viscosity is 8.7 × 10^−3^ Pa·s [23].

### 2.2. Fluid Analysis

The output of the model is the fluid pressure distributed at the sealing interface. The fluid pressure *p*(*r*) is governed by Reynolds equation [24], and average Reynolds equation [25] is usually applied to study fluid flow at the interface between the stator and the rotor. As shown in Figure 1b, the fluid film not only lubricates the interface surface and reduces friction and wear, but also supports the mating surface through the hydrodynamic effect. The average Reynolds equation is:

(1) $$F\frac{\partial }{\partial r}(\frac{rh_{n}^{3}}{12\mu }\frac{\partial D}{\partial r})+F\frac{1}{r}\frac{\partial }{\partial \alpha }(\frac{h_{n}^{3}}{12\mu }\frac{\partial D}{\partial \alpha })=\frac{r\omega }{2}[\frac{\partial h_{n}}{\partial \alpha }+(1-F)\frac{\partial (h_{n}D)}{\partial \alpha }]+r\frac{\partial h_{n}}{\partial t}$$

where *r* is the radial direction, *α* is the angular coordinate, *μ* is the dynamic viscosity of the lubricant, *ω* = ∂*α*/∂*t* is the relative angular speed, *h_n_* is the nominal film thickness and is related to the axial load of mechanical face seals. *D* is a universal variable that can be related to the fluid pressure *p* or the density *ρ*, depending on whether cavitation occurred or not. *F* is a switch function that allows us to describe whether cavitation occurs or not.

(2) $$F=0\begin{array}{cc} & D=\frac{\rho }{\rho_{0}}-1\begin{array}{cc} & p=p_{cav} \end{array} \end{array},F=1\begin{array}{cc} & D=p=p_{cav}\begin{array}{cc} & \rho =\rho_{0} \end{array} \end{array}$$

In this equation, *ρ* and *ρ*_0_ are the local and liquid densities, respectively, and *p_cav_* is the cavitation pressure. These parameters are assumed to be constant in the present study.

When preparing mathematical models, particular assumptions are made which include:

- This is a macro perspective, and the axial motion of mechanical face seal can be considered as a series of quasi-static process.
- When the axial-symmetric configuration is taken into account, then *∂p*/*∂α* = 0 and *∂h_n_*/*∂α* = 0.
- Installation misalignment is neglected.
- The lubricated fluid is incompressible and Reynolds equation is taken into account for smooth surfaces [26].
- Because of low heat generation, the influence of temperature change is ignored and constant viscosity of the medium is assumed.
- Mechanical seals usually work under mixed lubrication regime. The asperities interact with each other and are in a quasi-static process so that the squeeze term can be neglected [26] in the evaluation of the fluid pressure.

thus, Reynolds equation can be simplified as:

(3) $$\frac{\partial }{\partial r}(\frac{rh_{n}^{3}}{12\mu }\frac{\partial p}{\partial r})=0$$

Equation (3) is then integrated to obtain the fluid pressure *p*(r) as follows:

(4) $$p(r)=\frac{P_{o}-P_{i}}{\int_{r_{i}}^{r_{o}}\frac{1}{rh_{n}^{3}}dr}\int_{r_{i}}^{r}\frac{1}{rh_{n}^{3}}dr+P_{i}$$

where *p*(*r*) represents the distribution of pressure along the radial direction, the meaning the fluid pressure *F_f_* is:

(5) $$F_{f}=\int_{r_{i}}^{r_{o}}2\pi rp(r)dr$$

### 2.3. Asperity Contact Mechanics Analysis

In the case of boundary lubrication and mixed lubrication conditions, asperity contact occurs and the influence of asperity contact pressure on mechanical face seals cannot be neglected. Greenwood and Williamson proposed a model (G-W model) to calculate the asperity contact pressure [9]. In the G-W contact model, contact between two rough surfaces is reduced to that of contact between a smooth rigid surface and a rough surface. Independent Hertz contact is assumed to occur, as shown in Figure 1c. It is also assumed that the asperities have spherical summits, all with constant radius *R_e_*, and the asperity deformation is pure elastic deformation. According to the G-W model, the asperity contact pressure *p_ac_* is given by:

(6) $$p_{ac}(z,t)=\frac{W_{a}}{A_{n}}=\frac{4}{3}\eta_{e}R_{e}^{\frac{1}{2}}E_{e}\int_{\bar{h}(z,t)/\sigma_{e}}^{\infty }\phi (z){\left(z-\bar{h}(z,t)/\sigma_{e}\right)}^{\frac{3}{2}}dz$$

where *ϕ*(z) is the probability density function of the surface height distribution, $\overset{¯}{h}$(z,*t*) is the average value of film thickness, *η_e_* is the equivalent surface density of the asperities, *W*_a_ is the total asperity contact load, *A_n_* is the nominal contact area, and *E_e_* is the equivalent elastic modulus. The surface equivalent roughness *σ_e_* is defined as the root mean square roughness of stator and rotor surfaces heights [14].

The probability density function of the surface height distribution *ϕ*(z) is taken as:

(7) $$\phi (z)=\frac{1}{\sqrt{2\pi }\sigma_{e}}\exp(-\frac{z^{2}}{2\sigma_{e}^{2}})$$

Lebeck [7] proposed that the plausible value for mechanical contact pressure *p_c_* is the compressive yield stress *S_C_*. The contact interface portion of mechanical seal can be estimated by normal cumulative distribution function:

(8) $$b_{m}(r)=\int_{h}^{\infty }\phi (z)dz$$

The interfacial contact pressure can be expressed as:

(9) $$p_{c}(r)=b_{m}(r)S_{C}$$

Thus, the asperity contact load *F_c_* is given as:

(10) $$F_{c}=\int_{r_{i}}^{r_{o}}2\pi rp_{c}(r)dr$$

### 2.4. Mass-Spring-Damping Model

The mass-spring-damping model has two degrees of freedom: the axial motion and the circumferential rotation around the seal’s axis of the stator and rotor. Random vibration loading mainly affects the axial direction of the mechanical face seal. The following discussion focuses on an analysis of the axial force.

**Figure 2:**
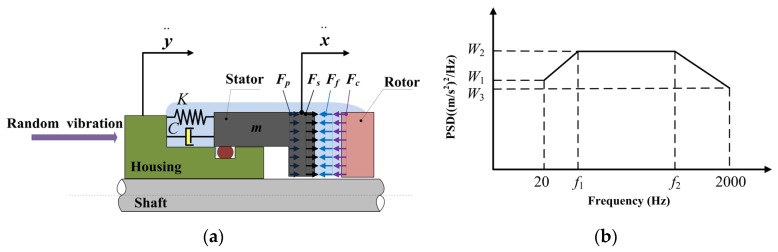
(**a**) Mass-spring-damping model; (**b**) acceleration power spectral density (PSD).

A schematic of the mass-spring-damping model is provided in Figure 2a. The mechanical face seal in the axial direction is abstracted as a mass-spring-damping system under random vibration loading. The housing is fixed to the pump and can be regarded as a moving base, while the O-ring produces the damping effect and the spring generates elastic force. A mass-spring-damping system with a discrete mass *m*, damping coefficient *C*, and spring stiffness *K* is placed on a moving base with acceleration $\ddot{y}$.

It is assumed that the mechanical seal is in a state of equilibrium in its initial state, that is, the closing force *F*_cl_ equals the opening force *F*_o_. The purpose of this is to facilitate the study of response characteristics of mechanical seals under random vibration.

The closing force *F*_cl_ consists of hydrostatic pressure *F*_p_ and seal spring force *F*_s_, $F_{p}=\pi (r_{o}^{2}-r_{b}^{2})P_{o}+\pi (r_{b}^{2}-r_{i}^{2})P_{i}$, and *F*_cl_ is given by:

(11) $$F_{cl}=\pi (r_{o}^{2}-r_{b}^{2})P_{o}+\pi (r_{b}^{2}-r_{i}^{2})P_{i}+F_{s}$$

The opening force *F*_o_ consists of fluid pressure *F_f_* and asperity contact load *F*_c_, and *F*_o_ is given by:

(12) $$F_{o}=F_{f}+F_{c}$$

In the initial state, the opening force balances the closing force:

(13) $$F_{cl}=F_{o}$$

In the case of random vibration loading input, in the axial direction of mechanical seal, $m\ddot{x}=\sum F$, that is:

(14) $$m\ddot{x}=C(\dot{y}-\dot{x})+K(y-x)+F_{e}$$

where *F*_e_ denotes force other than damping force and seal spring force, according to the principle of superposition, and the initial value of *F*_e_ is assumed to be zero. A relative displacement is then defined as:

(15) z=x−y

Substituting the relative displacement terms into Equation (14) provides:

(16) $$m\ddot{z}+C\dot{z}+Kz=-m\ddot{y}$$

By convention, $(C/m)=2\xi \omega_{n}$, $(K/m)=\omega_{n}^{2}$, where *ω_n_* is the natural frequency and *ξ* is the damping ratio. The final equation yields:

(17) $$\ddot{z}+2\xi \omega_{n}\dot{z}+\omega_{n}^{2}z=-\ddot{y}$$

The derivation is continued in Appendix A (Equations (A1)–(A18)). The excitation takes the form of a base input power spectral density. The root mean square (RMS) value of the response of the mass-spring-damping system can be expressed as:

(18) $${\ddot{x}}_{RMS}(f_{n},\xi )=\sqrt{\int_{0}^{\infty }[\frac{1+{\left(2\xi f/f_{n}\right)}^{2}}{{\left(1-{\left(f/f_{n}\right)}^{2}\right)}^{2}+{\left(2\xi f/f_{n}\right)}^{2}}]{\hat{Y}}_{aPSD}(f)df}$$

The acceleration power spectral density (PSD) of mechanical seals is shown in Figure 2b, as given by documents such as the MIL-STD-810G METHOD [27]. In Figure 2b, ascending spectrum is from 20 Hz to *f*_1_, flat spectrum is from *f*_1_ to *f*_2_, and descending spectrum is from *f*_2_ to 2000 Hz. The values of *f*_1_ and *f*_2_, *W*_1_, *W*_2_, and *W*_3_ are obtained from specific tests. It is common to use the 3-sigma (3σ) values of the acceleration responses for verification of the mechanical structure design. The calculated ${\ddot{x}}_{RMS}(f_{n},\xi )$ value is equal to 1σ value. In this work, it is assumed that the mean value of random vibration response spectrum is zero and is Gauss distribution with a probability density of:

(19) $$p(x)=\frac{1}{{\ddot{x}}_{RMS}(f_{n},\xi )\sqrt{2\pi }}\exp(-\frac{x^{2}}{2{\ddot{x}}^{2}{}_{RMS}(f_{n},\xi )})$$

The 3-sigma approach can be applied to analyze the lubrication state of mechanical face seals in which *F*_v_ denotes the equivalent force caused by random vibration loading, and can be expressed as:

(20) $$F_{v}=3{\ddot{x}}_{RMS}(f_{n},\xi )•m$$

Because of the existence of *F*_v_, the mechanical face seal is no longer in initial equilibrium state, *F*_v_ is the increase value of closing force *F*_cl_, and the opening force *F*_o_ also changes with it.

### 2.5. Duty Parameter

The duty parameter *G* was put forward by Stribeck following his investigation into bearing lubricity. Scholars including Sommerfeld, Gumbel, and Hershey have since applied this similarity number of friction characteristics in the field of sealing technology [28]. The duty parameter indicates the operating condition of mechanical seals and the magnitude of the hydrodynamic effect in the fluid film, that is, it could express the friction characteristic of mechanical seals. The duty parameter of mechanical seals is defined as the ratio of the viscosity force of the liquid film between the end faces and the closing force of the seal faces *F_cl_*:

(21) $$G=\frac{\mu \omega (r_{o}^{2}-r_{i}^{2})}{2F_{cl}}$$

A method in which friction regime is judged by the duty parameter *G* was put forward by Chen [29]. The relationship between the lubrication regime of mechanical seals and the duty parameter *G* is listed in Table 1.

### 2.6. Wear Model

In the present work, Archard wear model is applied to calculate the local wear rate [30]:

(22) $$V=\frac{K_{W}}{H}F_{W}L$$

where *V* is the volume loss, *L* is the sliding distance, *K_W_* is the dimensionless wear coefficient, *H* is the hardness of the stator, *F_W_* is the normal force, and wear is mainly caused by the asperity contact, so *F_W_* equals *F*_c_. Because the rotor is much harder than the stator, it is assumed that the wear only takes place on the stator surface.

The wear modulus *k_w_* is defined as *K_W_*/H, so the wear model can be rewritten as:

(23) $$V=k_{w}F_{W}L$$

The wear depth *h_w_* is given as:

(24) $$h_{w}=k_{w}\frac{F_{W}}{\pi (r_{o}^{2}-r_{i}^{2})}L$$

Wear modulus *k_w_* is commonly evaluated through experiments under specific lubricated conditions. When the shaft is rotating under random vibration loading, the lubrication conditions vary with time. The 3-sigma approach can be applied to calculate the *F_W_* of mechanical face seals under random vibration loading. The derivative of the loss of volume with respect to sliding distance is defined as the wear distance rate *k*_s_. Thus, the wear distance rate is given by [30]:

(25) $$k_{s}=\frac{dh_{w}}{dL}=k_{w}\frac{F_{W}}{\pi (r_{o}^{2}-r_{i}^{2})}$$

## 3. Numerical Algorithm

**Figure 3:**
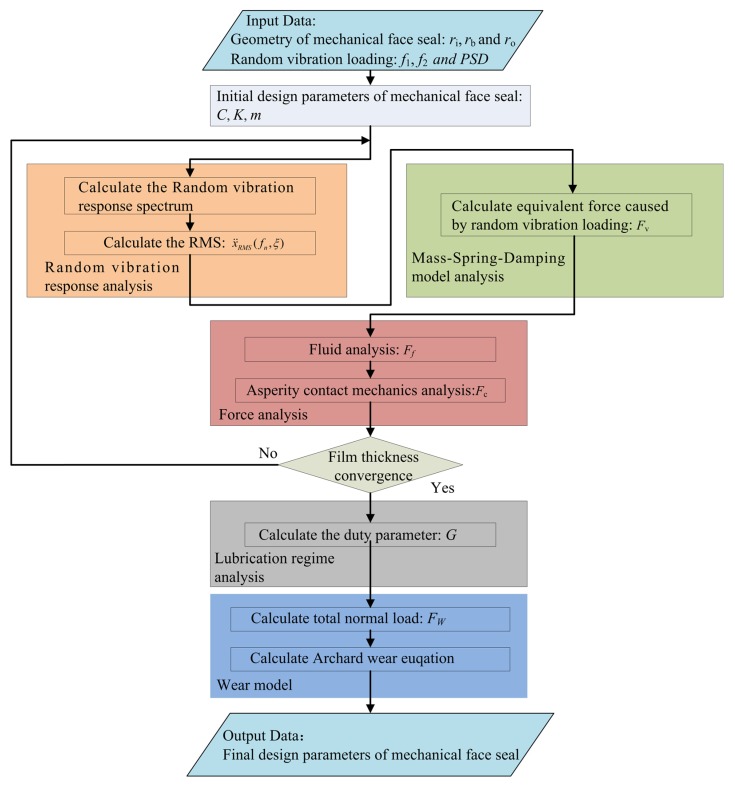
Flowchart of the proposed simulation method.

The analysis of mechanical face seals under random vibration loading based on the proposed model is performed according to the flowchart described in Figure 3.

The input data includes the geometric parameters of mechanical face seal and random vibration loading. In the proposed mathematical model, the calculation process begins with the initial design parameters *C*, *K*, and *m* of the mechanical face seal. It then proceeds with random vibration response analysis, mainly calculating the random vibration response spectrum and RMS. The next step is mass-spring-damping model analysis, which aims at calculating the equivalent force caused by random vibration loading. Force analysis is then conducted, mainly calculating the hydrodynamic force and asperity contact force. Random vibration response analysis, mass-spring-damping model analysis, and force analysis are iterated until the film thickness achieves convergence. This process is followed by lubrication regime analysis, with the main purpose to analyze the relationship between duty parameter *G* and random vibration loading. Finally, wear analysis is carried out, mainly calculating the total normal load and solving the Archard wear equation. In this paper, surface roughness is considered to be a constant, the purpose is to get the asperity contact load *F*_c_. The clearance between the stator and rotor determines the asperity contact load *F*_c_. Similarly, according to the Reynolds equation, this clearance also determines the fluid pressure *F_f_*. The change of stator surface height reflects the change of surface profiles. When the initial design parameters of the mechanical face seal cannot meet the operation requirements, new design parameters are re-introduced into the calculation process.

## 4. Numerical Experiments and Discussion

### 4.1. Random Vibration Loading

**Figure 4:**
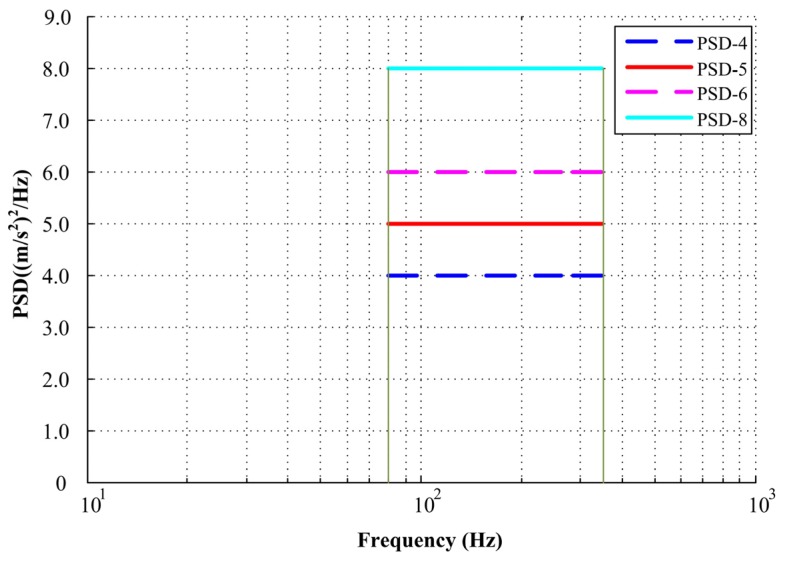
Flowchart of the proposed simulation method.

Random vibration loads can be conveniently described by PSD functions. According to the measured data in engineering, the most important part is the flat spectrum with a frequency from *f*_1_ to *f*_2_. In this work, *f*_1_ was 80 Hz and *f*_2_ was 350 Hz. The amplitude of input acceleration PSDs for simulation were 4.0, 5.0, 6.0, and 8.0 (m/s^2^)^2^/Hz, respectively, which represents the PSD value range of a mechanical seal in operation, as shown in Figure 4.

### 4.2. Random Vibration Response Spectrum

**Figure 5:**
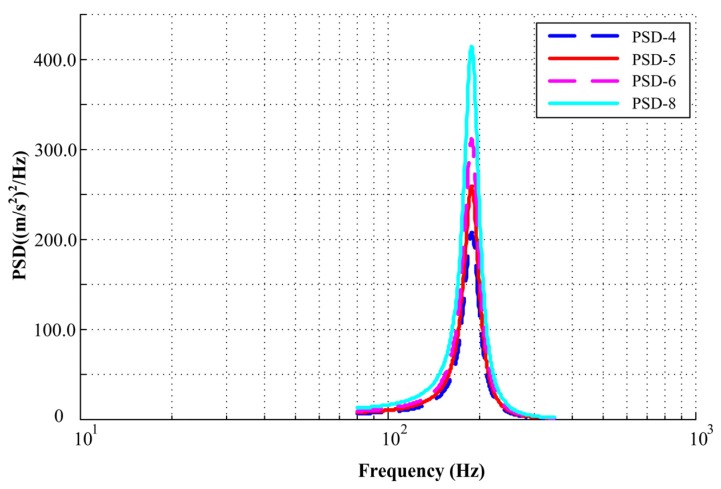
Random vibration response spectrum curve under different input acceleration PSDs.

The random vibration response spectra are all based on the response of the mass-spring-damping model exited to random accelerations. The probability that the response will exceed a critical level is of great significance. If the response levels exceed the allowable value, then severe wear will occur. As illustrated in Figure 5, with the increase of input acceleration PSDs, the random vibration response spectrum curves increase. The main parameter values in this section are set as shown in Table 2.

**Figure 6:**
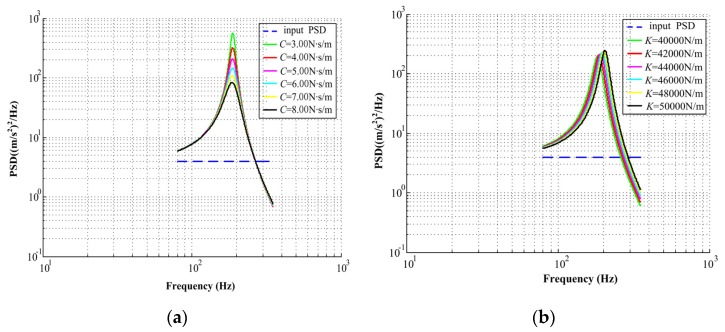
(**a**) Random vibration response curve under different axial damping; (**b**) random vibration response curve under different axial stiffness.

Under the same input acceleration PSD, the value is 4.0 (m/s^2^)^2^/Hz, and for different axial damping values, the random vibration response curve is shown in Figure 6a. When axial damping increases from 3.00 N∙s/m to 8.00 N∙s/m, the random vibration response curve has a downward trend, shown as a log-log coordinate system in the figure. It can thus be concluded that random vibration can be suppressed by increasing the damping.

### 4.3. Root Mean Square Acceleration Response

**Figure 7:**
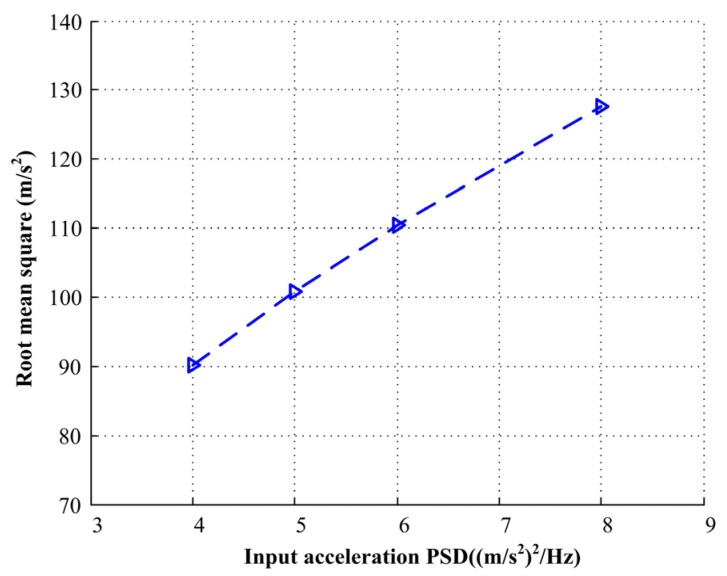
The root mean square (RMS) acceleration response under different input acceleration PSDs.

Random vibration response spectrum analysis determines the RMS response of the stator to an acceleration base input. In this work, random vibration responses were simulated effectively using Equation (18). The total RMS value is equal to the square root of the area bounded by the PSD function between the initiation frequency *f*_1_ and termination frequency *f*_2_. Figure 7 shows the RMS value ${\ddot{x}}_{RMS}(f_{n},\xi )$ of the acceleration response when the input PSDs are 4.0, 5.0, 6.0, and 8.0 (m/s^2^)^2^/Hz, respectively. According to Figure 7, it can be seen that the RMS value increases with the increase of input acceleration PSDs.

According to Equation (20), the corresponding equivalent force *F*_v_ caused by random vibration loading can then be calculated.

### 4.4. Force Analysis and Lubrication Regime Analysis

In the initial equilibrium state, the mechanical face seal works under mixed lubrication regime and the closing force *F*_cl_ is equal to the opening force *F*_o_, according to the design parameters of mechanical seal. The balance radius *r*_b_ is equal to 10.0 × 10^−3^ m, and the initial preload of the seal spring *F*_s_ is 25.0 N, so the closing force is 43.83 N.

The initial angular velocity is 200 rad/s, the dynamic viscosity is 8.7 × 10^−3^ Pa∙s, on the basis of the relationship between the lubrication regime of mechanical seals and the duty parameter *G* listed in Table 1. When *F*_cl_ is less than 31.95 N, the mechanical face seal works under full film lubrication regime and when *F*_cl_ is greater than 31.95 N and less than 638.93 N, it works under the mixed lubrication regime. When *F*_cl_ is greater than 638.93 N and less than 1597.32 N, it is under boundary lubrication regime. According to Equation (21), *G* is equal to 7.29 × 10^−^^7^, so the mechanical seal is operating under mixed lubrication regime.

**Figure 8:**
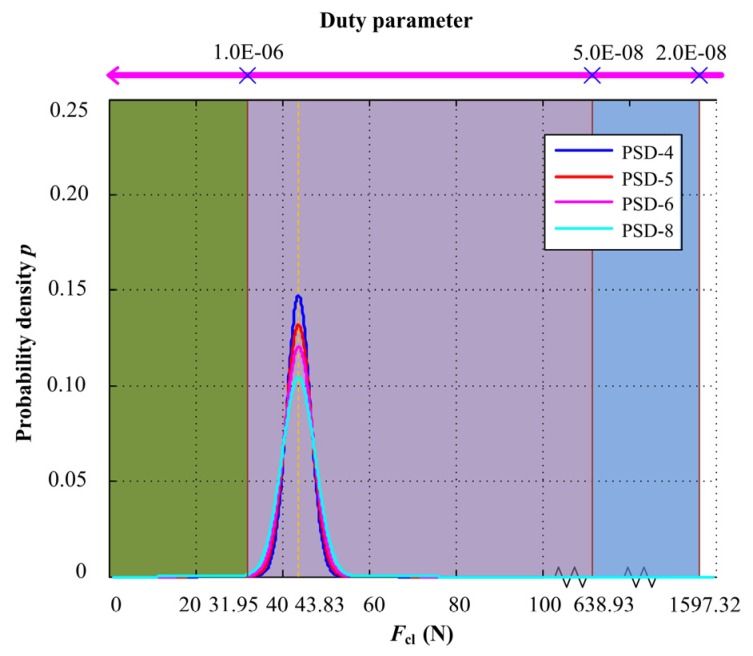
Probability density of closing force *F*_cl_ under different input acceleration PSDs and the relationship with duty parameter *G*.

**Figure 9:**
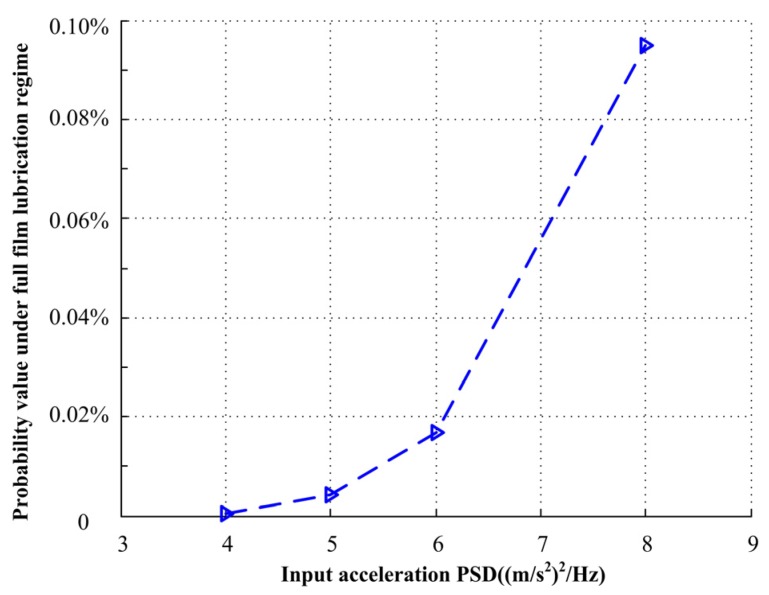
Probability value under different input acceleration PSDs.

When the input acceleration PSDs are 4.0, 5.0, 6.0, and 8.0 (m/s^2^)^2^/Hz, respectively, the probability density of closing force *F*_cl_ under different input acceleration PSDs and the relationship with duty parameter *G* is shown in Figure 8. The green area represents full film lubrication regime, the purple area represents mixed lubrication regime, and the blue area represents boundary lubrication regime. When the input acceleration PSDs are small, the mechanical face seal mainly works under mixed lubrication regime, and with the increase of input PSDs, the probability of the mechanical face seal working under full film lubrication regime increases, with the probability value shown in Figure 9. Because of the balance radius *r*_b_ and the initial preload of seal spring *F*_s_, the mechanical seal will not work under boundary lubrication regime.

**Figure 10:**
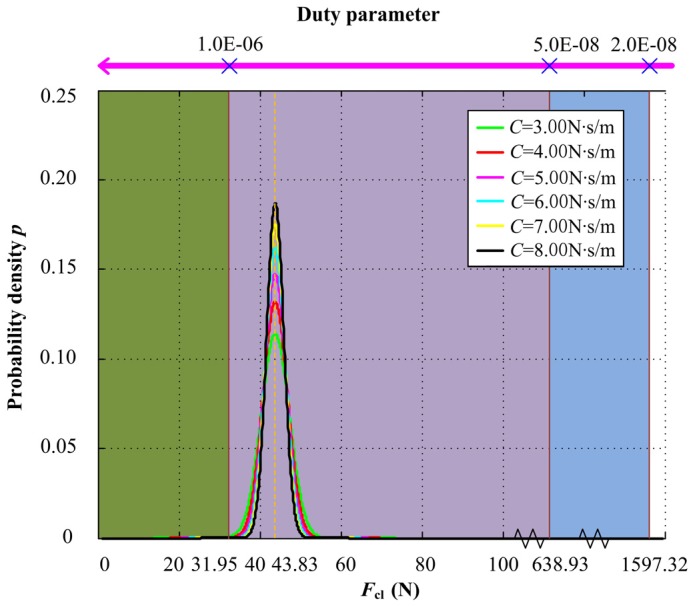
Probability density of closing force *F*_cl_ under different axial damping and the relationship with duty parameter *G*.

**Figure 11:**
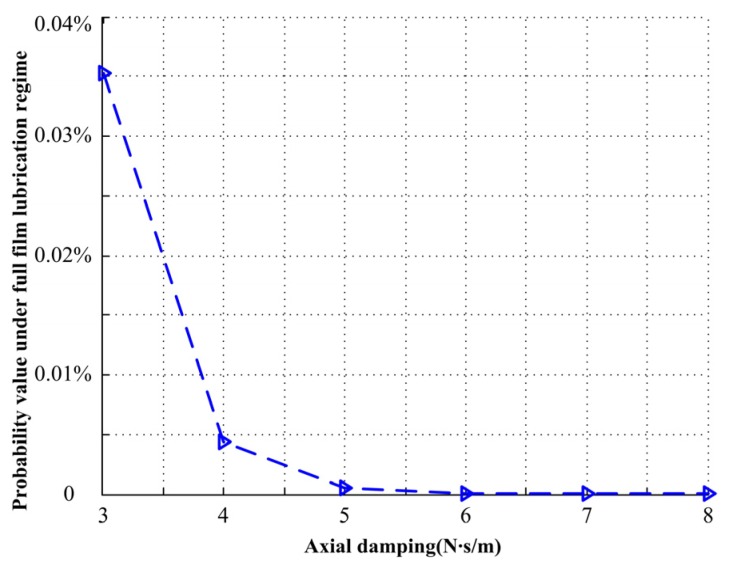
Probability value under different axial damping.

When axial damping increases from 3.00 N∙s/m to 8.00 N∙s/m, the input acceleration PSD is 4.0 (m/s^2^)^2^/Hz, with the probability density of closing force *F*_cl_ under different axial damping and the relationship with duty parameter *G* shown in Figure 10. The green area represents full film lubrication regime, the purple area represents mixed lubrication regime, and the blue area represents boundary lubrication regime. When the axial damping is large, the mechanical face seal mainly works under mixed lubrication regime, and with the decrease of axial damping, the probability of the mechanical face seal operating under full film lubrication regime increases, with the probability value shown in Figure 11.

**Figure 12:**
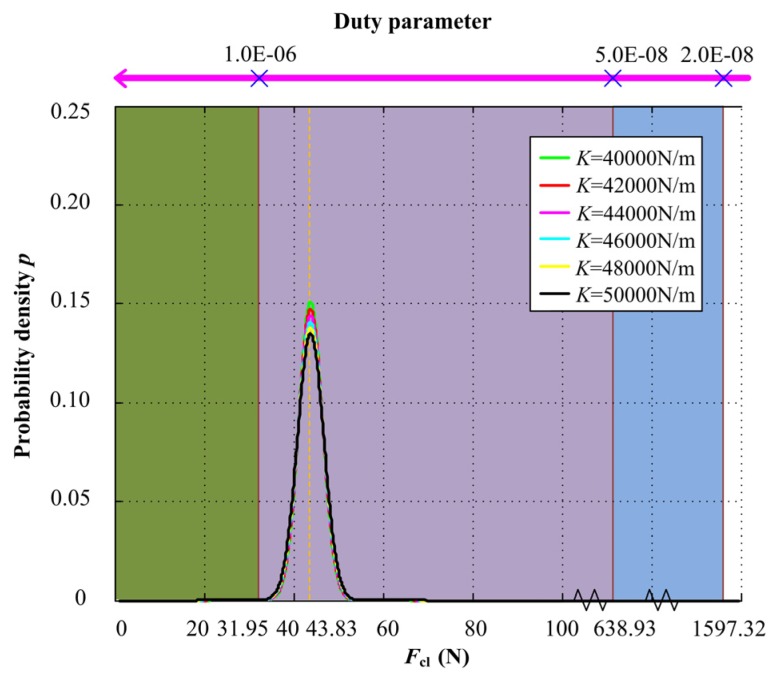
Probability density of closing force *F*_cl_ under different axial stiffness and the relationship with duty parameter *G*.

**Figure 13:**
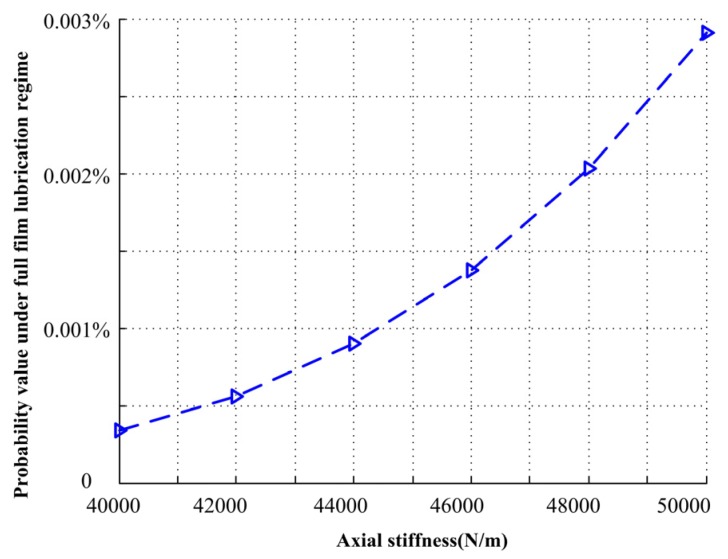
Probability value under different axial stiffness.

Similarly, when the input acceleration PSD is 4.0 (m/s^2^)^2^/Hz, for different axial stiffness values, the axial stiffness increases from 40000 to 50000 N/m. The probability density of closing force *F*_cl_ under different axial stiffness and the relationship with duty parameter *G* is shown in Figure 12. When the axial stiffness is small, the mechanical face seal mainly works under mixed lubrication regime, and with the increase of axial stiffness, the probability of the mechanical face seal operating under full film lubrication regime increases, but not obviously, and the probability value is shown in Figure 13.

### 4.5. Wear Analysis

According to Equations (24,25), it can be observed that the wear distance rate is directly related to the normal force *F_W_*, and its value is also equal to the asperity contact load *F*_c_. Here, wear modulus *k_w_* was set as 0.35 × 10^−6^ mm^3^/N∙m, which is obtained from wear testing. It was also assumed that the shaft is rotating at constant speed, which is 200 rad/s. The 3-sigma approach was then applied to analyze the equivalent force caused by random vibration loading.

**Figure 14:**
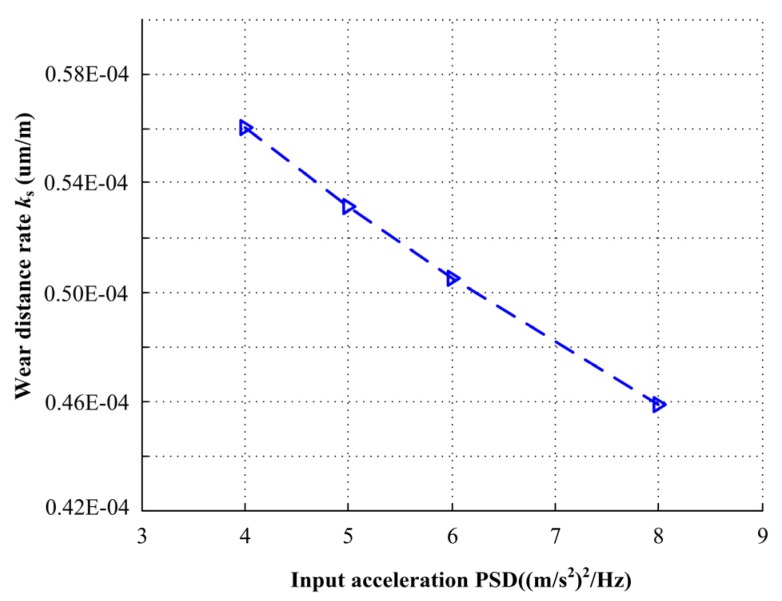
Wear distance rate under different input acceleration PSDs.

The relationship between the input acceleration PSDs and wear distance rate is shown in Figure 14. The wear distance rate decreases when the input acceleration PSDs improve. The wear distance rate decreases from 0.56 × 10^−4^ to 0.46 × 10^−4^ um/m with increase of the input acceleration PSDs from 4.0 to 8.0 (m/s^2^)^2^/Hz. With the increase of input PSDs, the probability of mechanical face seal under full film lubrication regime increases. Therefore, in practical application, the magnitude of input acceleration PSDs must be considered.

**Figure 15:**
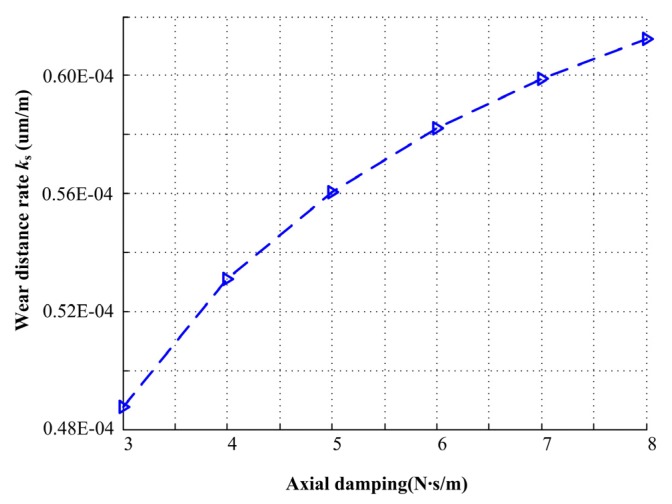
Wear distance rate under different axial damping.

The effect of mechanical face seal axial damping on wear distance rate is illustrated in Figure 15. The wear distance rate increases when the axial damping increases. The wear distance rate increases from 0.49 × 10^-4^ to 0.61 × 10^-4^ um/m with an increase of the axial damping from 3.0 to 8.0 N∙s/m. With the increase of axial damping, the probability of the mechanical face seal under full film lubrication regime decreases. Therefore, decreasing axial damping can restrain random vibration and reduce wear.

**Figure 16:**
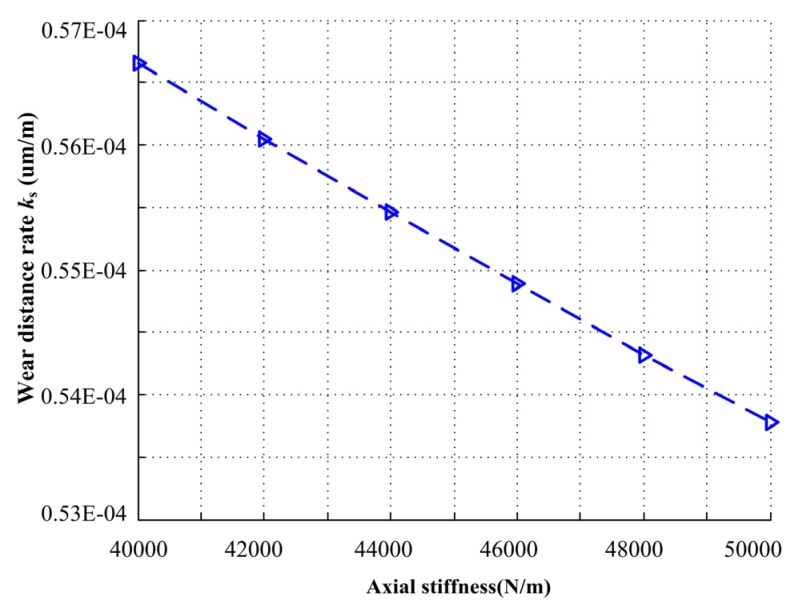
Wear distance rate under different axial stiffness.

The effect of axial stiffness on wear distance rate is illustrated in Figure 16. The wear distance rate decreases when the axial stiffness increases. The wear distance rate decreases from 0.567 × 10^−4^ to 0.538 × 10^−4^ um/m with an increase in the axial stiffness from 40000 to 50000 N/m. With higher axial stiffness, the probability of the mechanical face seal working under full film lubrication regime increases, but the effect is not significant. As a result of $(K/m)=\omega_{n}^{2}$, when axial stiffness changes from 40000 to 50000 N/m, for a determined mass *m*, the natural frequency range is from 183.8 to 205.5 Hz. In practical application, the frequency of this interval should be avoided to prevent resonance.

## 5. Conclusions

In this paper, mechanical face seals for hydraulic pumps used in air and space vehicles were studied. The effects of random vibration loading on seal lubrication regime and seal wear were focused. The mechanical face seal in the axial direction was abstracted as a mass-spring-damping system. The effects of input acceleration PSDs and dynamic parameters such as axial stiffness and axial damping on wear of the mechanical face seal were also taken into consideration. The model proposed in this paper can be used to analyze the lubrication regime of the mechanical face seal. Its purpose is to determine optimal mechanical specifications, reduce wear, and improve service life.

A simulation study was performed, and the results indicate that when the input acceleration PSDs are small, the mechanical face seal mainly works under mixed lubrication regime. With the increase of input acceleration PSDs, the probability of the mechanical face seal working under full film lubrication regime increases. The results also indicate that with the decrease of axial damping or the increase of axial stiffness, the probability of the mechanical face seal operating under full film lubrication regime increases.

The effects of the input acceleration PSDs, axial stiffness, and axial damping on wear were also studied. Numerical simulations demonstrated that increase in input acceleration PSDs results in an decreased wear distance rate. Similarly, an increase in axial damping or decrease in axial stiffness results in the increase of wear distance rate and the effect of axial damping is more significant.

The presented work provides a foundation for engineers to design mechanical face seals under random vibration loading. This research is carried out from the macroscopic perspective, and micromechanical aspects, such as the interaction of lubricant and asperities under random vibration loading can be further studied separately. Future work informed by the model presented in this article will include a comprehensive study of frictional heating, thermal deformation, the squeeze term in Reynolds equation, and viscosity variations in the film, aiming to reduce wear and improve the service life of mechanical face seals.

## Figures and Tables

**Table 1:** Relationship between lubrication regime and duty parameter *G*.

Lubrication Regime	Duty Parameter *G*
Full film lubrication regime	$G>1\times 10^{-6}$
Boundary lubrication regime	$2\times 10^{-8}<G<5\times 10^{-8}$
Mixed lubrication regime	$5\times 10^{-8}<G<1\times 10^{-6}$

**Table 2:** Main parameter values.

Parameter	Meaning	Value
*C*	Axial damping	5.00 N∙s/m
*K*	Axial stiffness	42000 N/m
*m*	Mass	0.03 kg
*r*_i_	Inner stator radius	9.3 × 10^−3^ m
*r_b_*	Balance radius	10.0 × 10^−3^ m
*r*_o_	Outer stator radius	11.1 × 10^−3^ m
*f*_1_	Initiation frequency	80 Hz
*f*_2_	Termination frequency	350 Hz
*σ_e_*	Surface roughness	0.152 × 10^−6^ m
*μ*	Dynamic viscosity	8.7 × 10^−3^ Pa∙s
*ω*	Angular velocity in working state	200 rad/s
*k*_w_	Wear modulus	0.35 × 10^−6^ mm^3^/N∙m

## Appendix A

(A1) $$\ddot{z}+2\xi \omega_{n}\dot{z}+\omega_{n}^{2}z=-\ddot{y}$$

Now taking the Fourier transform of each side

(A2) $$\int_{-\infty }^{\infty }\{\ddot{z}+2\xi \omega_{n}\dot{z}+\omega_{n}^{2}z\}e^{-j\omega t}dt=\int_{-\infty }^{\infty }\left\{-\ddot{y}\right\}e^{-j\omega t}dt$$

Note that the approach used here is rigorous. Let

(A3) $$Y(\omega )=\int_{-\infty }^{\infty }\{y(t)\}e^{-j\omega t}dt$$

(A4) $$X(\omega )=\int_{-\infty }^{\infty }\{x(t)\}e^{-j\omega t}dt$$

We can get,

(A5) $$\int_{-\infty }^{\infty }\{\dot{z}(t)\}e^{-j\omega t}dt=(j\omega )Z(\omega )$$

(A6) $$\int_{-\infty }^{\infty }\{\ddot{z}(t)\}e^{-j\omega t}dt=-\omega^{2}Z(\omega )$$

Definition

(A7) $$Z_{a}(\omega )=-\omega^{2}Z(\omega )=\int_{-\infty }^{\infty }\{\ddot{z}(t)\}e^{-j\omega t}dt$$

(A8) $$Y_{a}(\omega )=-\omega^{2}Y(\omega )=\int_{-\infty }^{\infty }\{\ddot{y}(t)\}e^{-j\omega t}dt$$

Recall (A2), by substitution,

(A9) $$-\omega^{2}Z(\omega )+j\omega (2\xi \omega_{n})Z(\omega )+\omega_{n}^{2}Z(\omega )=Y_{a}(\omega )$$

(A10) $$Z_{a}(\omega )=\frac{\omega^{2}Y_{a}(\omega )}{(\omega_{n}^{2}-\omega^{2})+2j\xi \omega \omega_{n}}$$

The relative acceleration equation can be expressed in terms of Fourier transforms as:

(A11) $$Z_{a}(\omega )=X_{a}(\omega )-Y_{a}(\omega )$$

(A12) $$X_{a}(\omega )=\frac{\left[\omega_{n}^{2}+2j\xi \omega \omega_{n}]Y_{a}(\omega \right)}{(\omega_{n}^{2}-\omega^{2})+2j\xi \omega \omega_{n}}$$

Multiply each side by its complex conjugate,

(A13) $$X_{a}(\omega )X_{a}^{*}(\omega )=\frac{\omega_{n}^{2}[\omega_{n}^{2}+{\left(2\xi \omega \right)}^{2}]Y_{a}(\omega )Y_{a}^{*}(\omega )}{{\left(\omega_{n}^{2}-\omega^{2}\right)}^{2}+{\left(2\xi \omega \omega_{n}\right)}^{2}}$$

The Fourier transforms are converted into power spectral densities,

(A14) $$\underset{T\to \infty }{\lim}\frac{1}{T}X_{a}(\omega )X_{a}^{*}(\omega )=X_{aPSD}(\omega )$$

(A15) $$\underset{T\to \infty }{\lim}\frac{1}{T}Y_{a}(\omega )Y_{a}^{*}(\omega )=Y_{aPSD}(\omega )$$

(A16) $$X_{aPSD}(\omega )=\frac{\omega_{n}^{2}[\omega_{n}^{2}+{\left(2\xi \omega \right)}^{2}]Y_{aPSD}(\omega )}{{\left(\omega_{n}^{2}-\omega^{2}\right)}^{2}+{\left(2\xi \omega \omega_{n}\right)}^{2}}$$

Equation (A16) can be transformed as a function of frequency *f* as follows:

(A17) $${\hat{X}}_{aPSD}(f)=\frac{\left[1+{\left(2\xi f/f_{n}\right)}^{2}]{\hat{Y}}_{aPSD}(f\right)}{{\left(1-{\left(f/f_{n}\right)}^{2}\right)}^{2}+{\left(2\xi f/f_{n}\right)}^{2}}$$

(A18) $${\ddot{x}}_{RMS}(f_{n},\xi )=\sqrt{\int_{0}^{\infty }[\frac{1+{\left(2\xi f/f_{n}\right)}^{2}}{{\left(1-{\left(f/f_{n}\right)}^{2}\right)}^{2}+{\left(2\xi f/f_{n}\right)}^{2}}]{\hat{Y}}_{aPSD}(f)df}$$
